# First Case of COVID-19-Associated Collapsing Glomerulopathy in Sub-Saharan Africa

**DOI:** 10.1155/2020/8820713

**Published:** 2020-09-28

**Authors:** Yannick M. Nlandu, Jean-Robert R. Makulo, Nestor M. Pakasa, Ernest K. Sumaili, Clarisse N. Nkondi, Justine B. Bukabau, François K. Beya, Nazaire M. Nseka, François B. Lepira

**Affiliations:** ^1^Kinshasa University Hospital, Nephrology Unit, Kinshasa, Democratic Republic of the Congo; ^2^Division of Uro-Nephropathology, Department of Pathology, Kinshasa, Democratic Republic of the Congo

## Abstract

Although the lungs remain the main target of SARS-CoV-2, other organs, such as kidneys, can be affected, which has a negative impact on the outcomes of COVID-19 patients. Although previous studies of kidney disease in COVID-19 reported mainly SARS-CoV-2-induced tubular and interstitial injury, there is growing evidence coming out of Africa of glomerular involvement, especially collapsing glomerulopathy seen particularly in people of African descent. We report a case of collapsing glomerulopathy revealed by acute kidney injury and a new onset of full blown nephrotic syndrome in a black Congolese patient coinfected with COVID-19 and malaria.

## 1. Introduction

The COVID-19 pandemic has caused unprecedented health, social, and economic crises worldwide [1, 2]. While the lungs are the organ primary target of SARS-CoV-2 and subsequent COVID-19, other organs, including kidneys, can be affected, and this is associated with increased mortality and morbidity [3, 4]. Previous autopsy reports from China and more recent reports from the USA have described mostly kidney tubular injury associated with SARS-CoV-2 [5, 6]. However, cumulating evidence from Europe and the USA yields collapsing glomerulopathy (CG), a variant of focal segmental glomerulosclerosis (FSGS) [7]. Interestingly, the patients reported in these early papers are of African descent, and the presence of two apolipoprotein L1 (*APOL1*) risk alleles likely contributes to the pathogenesis of the renal disease [7]. To date, there has been no similar report from continental Africa. We report the first COVID-19-associated CG from sub-Saharan Africa, in a Congolese patient infected with COVID-19, who presented with acute kidney injury (AKI) and de novo nephrotic syndrome.

## 2. Case Presentation

A 48-year-old African man presented to the Kinshasa University Hospital (KUH) for persistent fever since March 16, 2020, associated with dry cough, vomiting, myalgia, and generalized weakness. At home, he self-medicated with two antimalarial medications (artemether and lumefantrine) for three days. His symptoms persisted, and he visited a medical center on March 23, 2020. A blood smear demonstrated *Plasmodium falciparum* and was given intravenous Artesunate, another antimalarial drug, for five days combined with cefixime, a broad-spectrum, third generation cephalosporin.

Two weeks after his symptoms began, he experienced anuria and he visited the emergency unit of KUH on April 2, 2020. He stated that sixteen days previously he had been in contact with a person known to have COVID-19. His past medical history was remarkable for diabetes mellitus type II and hypertension for the past three years. His medications included the oral antidiabetic drug repaglinide and the antihypertensive drug amlodipine. His family history was notable in that his father had chronic kidney disease. Laboratory tests six months prior to admission included a serum creatinine of 0.72 mg/dL and urinalysis that showed no proteinuria.

On hospital admission, he was noted to be dyspneic, with a respiratory rate of 23 breaths per minute. His blood pressure was 152/84 mmHg and oxygen saturation was 93%. A nasopharyngeal swab sample was positive for COVID-19 mRNA by reverse transcriptase-PCR (RT-PCR). Kidney function tests revealed severe kidney dysfunction, with increased plasma level of urea (222 mg/dL) and creatinine (15.9 mg/dL). A kidney ultrasound excluded an obstructive uropathy; kidney echogenicity, length (13.2 cm), and resistive index (0.8) were increased. In the setting of anuria, no urinalysis was performed. Initial laboratory assessment and selected trends are depicted in Table 1.

A diagnosis of COVID-19 was made and the patient received chloroquine, azithromycin, and vitamin C, starting on day 1, according to the national COVID-19 management protocol (unpublished data). COVID-19 RT-PCR testing and *Plasmodium falciparum* blood smear performed on days 14 and 21 after his hospital admission were negative.

Assessment of kidney function included anuria, metabolic acidosis, hyperkalemia, and stage 3 AKI according to Kidney Disease Improving Global Outcomes (KDIGO) criteria. On April 2, the patient started hemodialysis three sessions per week. After five dialysis sessions, the patient progressively recovered renal function, with diuresis reaching 4 liters/day and a fall in serum creatinine levels (2 mg/dL). The clinical diagnosis was acute tubular necrosis.

**Figure 1:**
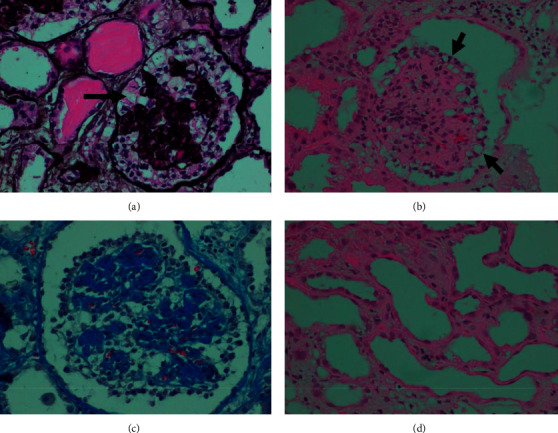
Kidney biopsy: representative light microscopy shows global collapse of the glomerular capillary loops accompanied by hyperplasia and hypertrophy of overlying glomerular epithelial cells (a–c) many of which contain scattered eosinophilic protein droplets (a, arrow) and numerous intracytoplasmic vacuoles (b, arrows). a: Jones methenamine silver stain; b: H&E stain; c: Masson's trichrome stain, ×400. Acute tubular injury is manifest in many cortical tubules, accompanied by interstitial edema and patchy interstitial mononuclear inflammatory infiltrates (d: H&E stain, ×400).

One week after recovery of kidney function (admission day 27), he developed full blown nephrotic syndrome (NS), characterized by heavy proteinuria (18 g/d), hypoalbuminemia (serum albumin 2.3 g/dL), and dyslipidemia; microscopic urinalysis was normal. He experienced progressive increase in serum creatinine up to 13 mg/dL. There were no episodes of hypotension, and the patient received no nephrotoxic drugs. A kidney ultrasound excluded renal vein thrombosis. Serologic analyses for hepatitis B, hepatitis C, cytomegalovirus, and HIV were negative. A percutaneous kidney biopsy was performed on hospital day 30. The biopsy contained three cores, each of which contained with both renal cortex and medulla. They yielded a total of 17 glomeruli, none of which was globally sclerotic. All but one displayed segmental or global collapse, implosion of the glomerular capillary tuft, and enlargement of Bowman space (Figure 1).

Bowman space was occupied by hypertrophic and hyperplastic epithelial cells in 1–3 layers some of which showed cytoplasmic vacuolization and occasional protein droplets. Multiple early synechiae connecting glomeruli to Bowman capsule were seen. There was a severe tubular injury, with degenerative and regenerative changes. They included epithelial cell denudation loss and attenuation of the tubular brush border and cellular blebbing and mitoses. These changes in tubules were strongly Ki-67 positive, a molecular marker of cell proliferation (not shown) by immunohistochemistry.

Tubules showed microcystic dilatation and contained positive proteinaceous casts. Some proximal tubules also contained abundant PAS-positive resorption protein droplets. Tubulitis was not seen. The interstitium showed mild edema and patchy moderate fibrosis and patchy moderate to marked chronic inflammatory cell infiltrate. The arteries and arterioles showed no conspicuous changes. The biopsy findings were interpreted as being consistent with the collapsing variant of FSGS based on Columbia classification of FSGS, also known as collapsing glomerulopathy (CG) [8].

**Figure 2:**
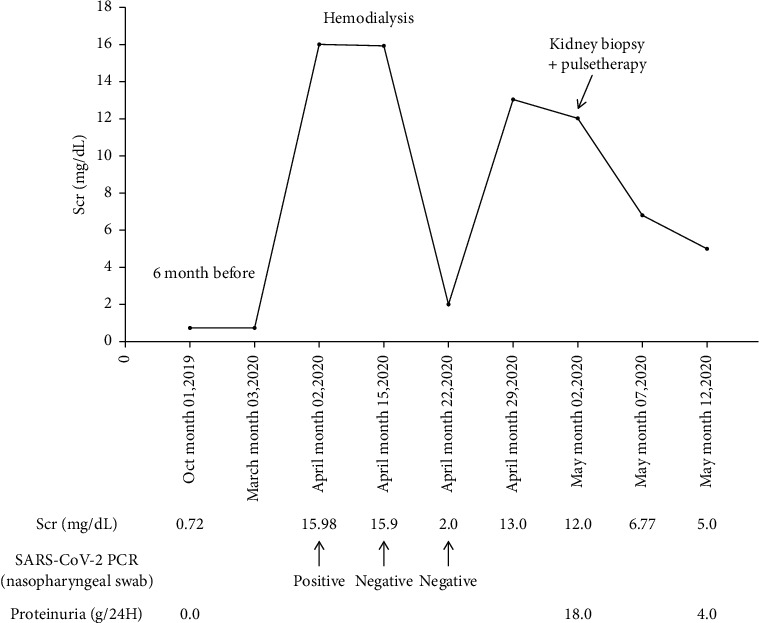
Changes in serum creatinine and proteinuria with time in a 48-year-old African male admitted for acute kidney injury with COVID-19.

Shortly after the kidney biopsy, the patient began producing urine. The patient received pulse therapy with methyl prednisolone intravenously, 500 mg on days 1, 2, and 3, and cyclophosphamide intravenously, 500 mg day 1; despite this therapy, proteinuria remains still heavy (4 g per day).  Figure 2 shows changes in serum creatinine and proteinuria with time. Although kidney function does not return to normal, serum creatinine levels decreased to 5.0 mg/dL after two weeks.

## 3. Discussion

Prior studies on kidney disease, based on postmortem autopsy specimens in COVID-19 patients, showed acute tubular injury without glomerular abnormalities [5]. The authors linked these observations to the fact that angiotensin converting enzyme type 2 (ACE2) and transmembrane protein serine type 2 (TMPRSS2), proteins required for SARS-CoV-2 entry and replication in host cells, are prominently expressed in epithelial cells of proximal convoluted tubes [9]. However, recent reports from COVID-19 patients demonstrated the glomerular injury mainly in the form of CG encountered particularly in people of African ancestry [7]. CG is an aggressive variant of FSGS exhibiting high rates of podocyte injury and depletion. Diseased podocytes exhibit a loss and gain of markers of differentiation and proliferation, respectively, and podocytes have been described to “transdifferentiate” toward a macrophage-like cell [10]. By light microscopy, CG is characterized by hyperplasic and hypertrophic visceral epithelial cells overlying segmentally or globally collapsed glomerular capillaries that are narrowed or obliterated by wrinkling and retraction of glomerular basement membranes [11]. The tubulointerstitial compartment often contains an infiltrate of mononuclear cells. Tubular epithelial cells typically display degenerative and regenerative changes and aberrant cellular proliferation and differentiation, leading to microcystic transformation. Tubular atrophy and interstitial fibrosis are common [11]. This histologic description corroborates the findings in the patient herein reported. Ancillary techniques such as electron microscopy (EM), immunofluorescence, in situ hybridization, and other sophisticated techniques have been used to identify pathogenic viral particles in biopsies from kidney structures in well-resourced settings, yielding so far no conclusive results besides podocyte changes related to proteinuria [12–14], although a recent EM report of a necropsy claims ultrastructural evidence for the presence of virus in podocytes [15].

In the present case report, AKI and NS with a bland microscopic urinalysis were the clinical expression CG. This clinical presentation of CG in the context of COVID-19 illness has been recently reported [11-17]. CG can be of idiopathic origin or most commonly secondary to autoimmune diseases, interferon therapy, and viral illnesses, including HIV, cytomegalovirus, and parvovirus B19 [16]. Our previous experience also reported CG associated with filariasis [17]. As many of these secondary causes were ruled out in the present patient, COVID-19 and coinfection with malaria were retained as potential causes of CG and ensuing NS and AKI. To date, less than 30 cases of CG have been described worldwide in the context of COVID-19, while AKI alone occurs in 1.3% to 36.4% [18]. Although rare, some cases of CG associated with malaria have also been described; indeed, malaria can be associated with hemolytic uremic syndrome or hemophagocytic syndrome [19, 20].

Kidney involvement in COVID-19 is now believed to originate from a double hit mechanism thought to rely upon the interaction of environmental factors, mainly SARS-CoV-2, altered host immune response, and genetic susceptibility. Recent data indicate that kidney injury in COVID-19 could be initiated by viral cytotoxicity or cytokine storm related to sepsis or other associated comorbidities [21]. Direct infection of kidney resident cells is enabled by the high expression of ACE2 and TMPRSS2 in epithelial cells of proximal convoluted tubes and podocytes [9]. The virus likely gains access to the kidney via the bloodstream [21].

Reports from Europe and the United States of America on CG in COVID-19 patients have indicated a high susceptibility of people of African descent compared to other people [7]. This high susceptibility of black people to CG has been reported to be linked to the presence of high kidney risk genotypes of *APOL1* genes, well-known risk factors for the development of CG in HIV and non-HIV black patients [22]. Regardless of the associated disease, nearly 70% of these African-descent patients with CG are homozygous for *APOL1* risk alleles [23]. In the Democratic Republic of the Congo, G1 *APOL1* risk variants are frequent and are associated with hypertension-attributed nephropathy [24]. High-risk *APOL1* genotype may mediate podocyte damage via up regulation of *APOL1* through activation of a viral program in the podocyte, leading to dysregulated endosomal trafficking and autophagic flux, resulting in podocyte depletion and glomerular scarring [11].

In conclusion, this first report from African continent confirms that collapsing glomerulopathy now referred to as collapsing-associated nephropathy (COVAN) represents an emerging entity in the context of COVID-19 pandemic, particularly involving individual with sub-Saharan African ancestry.

## Figures and Tables

**Table 1:** Summary of laboratory evaluations and relevant trends during hospitalization.

Test	Reference	March 16, 2020	April 2, 2020	April 15, 2020	April 22, 2020	April 29, 2020	May 2, 2020	May 7, 2020
Blood urea (mg/dl)	11.3–40.3		222.4	108.3	99.1	198.8	138	195.9
Serum creatinine (mg/dl)	0.6–1.3		15.98	15.9	2	13	12	6.77
Potassium (mmol/l)	3.0–5.0		5.2	4.7		3.7		
Sodium (mmol/l)	135–145		135	135		133		
Bicarbonate (mmol/l)	18–24		14.1			22		
Ionized calcium (mmol/l)	1.18–1.32		0.92	1.07		0.72		
Hemoglobin (g/dl)	12.5–15		18.3			14.3	11.1	11.4
Hematocrit (%)	36–45		50.1			42	33	31.9
White blood cell count (elements/mm^3^)	4,000–10,000		14,630					7,830
Lymphocyte count (%)	20–40		17.1					9.5
Platelets (elements/mm^3^)	150,000–450,000		313,000					366,000
Albumin (g/dl)	35–45						2.3	
Total cholesterol(mg/dl)	<200			22.06			384	
HDL cholesterol (mg/dl)	>45			840			51	
LDL cholesterol (mg/dl)	<100						272	
Triglycerides (mg/dL)	<150						307	
Erythrocyte sedimentation rate (mm/h)	10–20		85					
CRP (mg/l)	<6	>16					<6	
